# Delirious episode secondary to rotigotine: the psychotic patch

**DOI:** 10.1192/j.eurpsy.2023.1303

**Published:** 2023-07-19

**Authors:** M. A. Andreo Vidal, M. Calvo Valcárcel, P. Martínez Gimeno, P. Pando Fernández, B. Rodríguez Rodríguez, N. Navarro Barriga, M. Fernández Lozano, M. J. Mateos Sexmero, T. Jiménez Aparicio, M. D. C. Valdecillo Adame, C. de Andrés Lobo, G. Guerra Valera, M. Queipo de Llano de la Viuda, A. A. Gonzaga Ramirez, M. D. L. Á. Guillén Soto, A. Aparicio Parras, M. Esperesate Pajares

**Affiliations:** 1 Psiquiatría; 2 Psicología, Hospital Clínico Universitario de Valladolid, Valladolid, Spain

## Abstract

**Introduction:**

There is a fine line separating psychiatry and neurology. Most movement disorders can have psychiatric symptoms, not only those caused by the disease itself, but also those induced by the drugs used to treat them.

**Objectives:**

Presentation of a clinical case about a patient diagnosed with Parkinson’s disease presenting a several-month-long delirious episode due to dopaminergic drugs.

**Methods:**

Literature review on drug-induced psychosis episodes in Parkinson’s disease.

**Results:**

A 57-year-old patient with diagnosis of Parkinson’s disease for six years, who went to the emergency room accompanied by his wife due to delirious ideation. He was being treated with levodopa, carbidopa and rasagiline for years, and rotigotine patches whose dosage was being increased over the last few months.

His wife reported celotypical clinical manifestations and multiple interpretations of different circumstances occurring around her. He chased her on the street, had downloaded an app to look for a second cell phone because he believed she was cheating on him, and was obsessed with sex. He had no psychiatric background. It was decided to prescribe quetiapine.

The following day, he returned because he refused to take the medication since he thought he was going to be put to sleep or poisoned. It was decided to admit him to Psychiatry.

During the stay, rasagiline and rotigotine were suspended. Olanzapine and clozapine were introduced, with behavioral improvement and distancing from the psychotic symptoms which motivated the admission. The patient was also motorically stable. Although levodopa is best known for causing psychotic episodes, the symptons were attributed to rotigotine patches for temporally overlapping the dose increase.

**Conclusions:**

Psychiatric symptoms are the third most frequent group of complications in Parkinson’s disease after gastrointestinal complications and abnormal movements. All medication used to control motor disorders can lead to psychosis, not only dopaminergics, but also selegiline, amantadine and anticholinergics.

Excessive stimulation of mesocortical and mesolimbic dopaminergic pathways can lead to psychosis, which is the most common psychiatric problem related to dopaminergic treatment.

In the face of a psychotic episode, antiparkinsonian drugs which are not strictly necessary for motor control should be withdrawn. If this is not sufficient, levodopa dose should be reduced, considering the side effects that may occur. When the adjustment of antiparkinsonian treatment is not effective, neuroleptics, especially quetiapine or clozapine, should be administered. In a recent study, pimavanserin, a serotonin 5-HT2 antagonist, was associated with approximately 35% lower mortality than atypical antipsychotic use during the first 180 days of treatment in community-dwelling patients.

Medication should always be tailor-made to suit each patient and we usually have to resort to lowering or withdrawing the dopaminergic medication.

**Disclosure of Interest:**

None Declared

